# Undercarboxylated osteocalcin has no adverse effect on endothelial function in rabbit aorta or human vascular cells

**DOI:** 10.1002/jcp.30048

**Published:** 2020-09-16

**Authors:** Alexander Tacey, Sophie Millar, Tawar Qaradakhi, Cassandra Smith, Alan Hayes, Susan Anderson, Anthony Zulli, Saoirse O'Sullivan, Itamar Levinger

**Affiliations:** ^1^ Institute for Health and Sport Victoria University Melbourne Victoria Australia; ^2^ Australian Institute for Musculoskeletal Science (AIMSS) The University of Melbourne and Western Health St Albans Victoria Australia; ^3^ Division of Medical Sciences and Graduate Entry Medicine, School of Medicine, Royal Derby Hospital University of Nottingham Derby UK

**Keywords:** cardiovascular disease, cell culture techniques, hyperglycaemia, immunohistochemistry, osteocalcin

## Abstract

Undercarboxylated osteocalcin (ucOC) improves glucose metabolism; however, its effects on endothelial cell function are unclear. We examined the biological effect of ucOC on endothelial function in animal models ex vivo and human cells in vitro. Isometric tension and immunohistochemistry techniques were used on the aorta of male New Zealand white rabbits and cell culture techniques were used on human aortic endothelial cells (HAECs) to assess the effect of ucOC in normal and high‐glucose environments. Overall, ucOC, both 10 and 30 ng/ml, did not significantly alter acetylcholine‐induced blood vessel relaxation in rabbits (*p* > .05). UcOC treatment did not cause any significant changes in the immunoreactivity of cellular signalling markers (*p* > .05). In HAEC, ucOC did not change any of the assessed outcomes (*p* > .05). UcOC has no negative effects on endothelial function which is important to reduce the risks of off target adverse effects if it will be used as a therapeutic option for metabolic disease in the future.

## Graphical abstract

Undercarboxylated osteocalcin (ucOC) improves glucose metabolism; however, its effects on endothelial cell function are unclear. We examined the biological effect of ucOC on endothelial function in animal models ex vivo and human cells in vitro. We found that ucOC has no negative effects on endothelial function which is important to reduce the risks of off‐target adverse effects if it will be used as a therapeutic option for metabolic disease in the future.

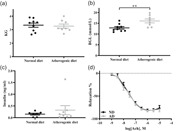

## 1 INTRODUCTION

The link between diabetes‐associated hyperglycaemia and the development of cardiovascular disease is well established (Ebong et al., 2013). Hyperglycaemia is a major independent risk factor for the development of atherosclerosis and vascular disease (Bornfeldt & Tabas, 2011; Rask‐Madsen & King, 2013). Exposure of endothelial cells to high glucose levels perturbs cell homeostasis, and an imbalance of biochemical pathways contributes ultimately to endothelial dysfunction (Bakker, Eringa, Sipkema, & van Hinsbergh, 2009). This imbalance causes several pathological effects, principally, the inability of the endothelium to regulate vasodilation and vasoconstriction (Bonetti, Lerman, & Lerman, 2003; Cahill & Redmond, 2016; Lerman & Zeiher, 2005).

Recent advances in the understanding of bone physiology have established bone as an active endocrine organ. Osteocalcin (OC) in its undercarboxylated form (ucOC) plays a role in glucose regulation and energy metabolism (Levinger et al., 2017; Li, Zhang, Yang, Li, & Dai, 2016). ucOC has been linked to enhanced secretion of insulin from pancreatic beta cells, improvements in insulin sensitivity, and regulation of glucose homeostasis (Ferron, Hinoi, Karsenty, & Ducy, 2008; Lin et al., 2017; Oury et al., 2011). Given these bioactive effects, it has been suggested that ucOC be targeted as a therapeutic treatment for metabolic diseases, including diabetes (Villafan‐Bernal, Sanchez‐Enriquez, & Munoz‐Valle, 2011). However, some studies report that OC may be associated with endothelial dysfunction and atherosclerosis. For example, in men and women with diabetes, lower levels of circulating total OC (tOC) have been associated with increased pulse wave velocity, intima–media thickness (IMT), and vascular complications (Q. Guo et al., 2017; Kanazawa et al., 2009). On the other hand, higher levels of tOC have also been associated with increased plaque development and IMT men and women with diabetes (Kanazawa et al., 2011; Reyes‐Garcia et al., 2012). Overall, the evidence is conflicting and the exact role of tOC and ucOC in the vasculature is unclear (Millar, Patel, Anderson, England, & O'Sullivan, 2017; Tacey et al., 2018). One major limitation of previous studies is that they only examined the serum levels of circulating tOC, and not its individual forms, in particular ucOC. Given the bioactivity of ucOC, its direct effect on blood vessels must be explored before any use as a therapeutic option for hyperglycaemia.

The aim of the current study was to determine (a) whether ucOC has an effect on endothelium‐dependent and endothelium‐independent vasodilation in rabbit aorta following incubations in normal and high glucose solutions, and (b) whether the treatment of human aortic endothelial cells (HAECs) with ucOC alters endothelial cell homeostasis following incubation in high glucose media.

## 2 METHODOLOGY

### 2.1 Animals

Male New Zealand White rabbits were housed in individual cages on a 12‐h light/dark cycle at 21°C, with access to water and standard chow diet ad libidum. At 12 weeks of age, the rabbits were randomised onto a normal chow diet (Guinea pig and rabbit pellets) or an atherogenic diet (a normal diet combined with 1% methionine, 0.5% cholesterol and 5% peanut oil (#SF00‐218) for 4 weeks (Zulli & Hare, 2009). At the completion of the 4‐week diet, the rabbits were weighed and then sedated (0.25 mg/kg medetomidine) and anaesthetised (4% isoflurane) before exsanguination via severing of the inferior vena cava. A random blood glucose sample was obtained from the inferior vena cava immediately upon exsanguination and was recorded with a blood glucose monitor (Freestyle Optimum Neo). A serum sample was obtained from the inferior vena cava to determine insulin concentration. Insulin was measured using an enzyme‐linked immunosorbent assay (ELISA) Kit and was completed according to the manufacturer's instructions (#90186; Australian Biosearch). This study was approved by the Victoria University Animal Ethics Committee (#14/005) and complied with the Australian National Health and Medical Research Council code for the care and use of animals for scientific purposes (8th edition).

### 2.2 Isometric tension myography

The abdominal aorta (immediately before the iliac bifurcation) was dissected and placed in ice‐cold Krebs solution ([mM] 118 NaCl, 4.7 KCl, 1.2 MgSO_4_·7H_2_O, 1.2 KH·2PO_4_, 25 NaHCO_3_, 11.7 glucose, and 1.25 CaCl). The aorta was cleaned of connective tissues, cut into rings (2–3 mm) and placed in individual organ baths containing Krebs warmed to 37°C and bubbled with 95% O_2_/5% CO_2_. This was followed by 30‐min acclimatisation. Blood vessel reactivity was measured via an isometric tension organ bath system (Zultek Engineering), as previously described (El‐Hawli et al., 2017; R. M. Smith, Rai, Kruzliak, Hayes, & Zulli, 2019). In brief, the aortic rings were carefully mounted on parallel hooks (one of which was connected to a force transducer) and stretched to a basal tension twice over a 1‐h period. Thereafter, the vessels were incubated for 2 h in normal Krebs solution (11.7 mM glucose) or high‐glucose Krebs solution (20 mM glucose) which has previously shown to cause a reduction in endothelium‐dependent vasodilation (X. Guo, Liu, Chen, & Guo, 2000; Taylor & Poston, 1994). Each organ bath was refreshed every 30 min with its respective Krebs solution. Aortic rings were constricted with phenylephrine (3 × 10^−7^ M) until a plateau occurred followed by a 5‐min incubation with either 10 or 30 ng/ml ucOC (Glu13, 17, 20, osteocalcin [1–46] [mouse] trifluoroacetate salt [H‐6552.0500; Auspep]) or control solution [Krebs]. The concentration of ucOC administered to each aortic ring was chosen based on physiological ranges (Hiam et al., 2019). Blood vessel reactivity was determined via cumulative dose‐response curves to the endothelium‐dependent vasodilator acetylcholine (ACh) or with the endothelium‐independent vasodilator sodium nitroprusside (SNP) in half‐log increments (10^−8^ to 10^−5^ M). The response of the vessels was measured on a software program (MEDIDAQ) which displays the tension of the vessel in grams. The log dose of ACh/SNP that produced the maximal relaxation was indicated by the *E*_max_ and the EC_50_ as the log dose that produced 50% of the *E*_max_. The area under the curve (AUC) was determined as the total area of relaxation below the phenylephrine plateau.

### 2.3 Immunohistochemistry

Following the isometric testing, aortic rings were immediately placed into 4% paraformaldehyde, left overnight, and then transferred into 1X phosphate‐buffered saline at 4°C. This was followed by paraffin processing (Microm STP120) and embedding in paraffin blocks. Sections were cut at 5 μm, deparaffinised in xylene, rehydrated and blocked with 1% goat serum in 10 mm Tris‐Cl (pH 7.4) for 20 min. Primary mouse monoclonal antibodies anti‐3‐nitrotyrosine [39B6] (#61392; Abcam) and endothelial nitric oxide synthase (eNOS) type III (#610296; BD Biosciences), p‐protein kinase B (Akt) 1/2 [Ser473] (NOVNB10056749; Novus Biologicals) and p‐mammalian target of rapamycin (mTOR) [59. Ser 2448] (SANTSC‐293133; Santa Cruz Biotechnology) at 1:100 dilution were applied overnight. Samples were also prepared where the primary antibody was omitted from the solution as a negative control. Samples were subsequently incubated with antimouse immunoglobulin G for 1 h (Immpress HRP Reagent Kit MP‐7452; Vector Laboratories). Diaminobenzidine (#550880; BD Biosciences) was applied as a chromogen before counterstaining with hematoxylin, dehydration, and mounting in dibutylphthalate polystyrene xylene (Arora, Hare, & Zulli, 2012). All chemicals and reagents were supplied by Sigma‐Aldrich unless otherwise specified.

### 2.4 Immunohistochemistry quantification

Images of each aortic ring were taken at ×40 magnification (Leica DFC 450F; Leica Microsystems). The endothelium was traced and the degree of brown immunoprecipitate (indicative of positive antigenic sites) was quantified using the MCID programme (MCID 7.0; Interfocus), as previously described (Qaradakhi et al., 2017; Zulli et al., 2006). Researchers were blinded to the samples for quantification. The proportional intensity (arbitrary unit) of brown immunoprecipitate was calculated as a ratio of colour intensity to proportional area, normalised to the negative control. Finally, the immunoreactivity of each protein was calculated based on a fold change from the respective control vessel (the control ring from the normal diet or atherogenic diet groups).

### 2.5 Cell culture

HAECs were purchased from PromoCell and maintained in commercial endothelial cell media with supplements (PromoCell) containing 1% penicillin–streptomycin (Sigma‐Aldrich) in a humidified incubator (5% CO_2_, 37°C), as previously established (Millar, Zala, Anderson, & O'Sullivan, 2019). Cells were used for experiments at passages 4 and 5. Cells were treated with either 5.6 mM normal glucose media (NG) or 16 mM high glucose media (HG) for 7 days with or without ucOC (10 ng/ml) to induce endothelial dysfunction. Media and cell lysates were collected at the end of the experiments. Each experiment was repeated independently three times. Human uncarboxylated osteocalcin (ucOC; amino acids 1–49, [Glu17, 21, 24]) was purchased from US Biological (O8060‐09C‐USB). d‐(+)‐glucose was purchased from Sigma‐Aldrich (#I9278 and #G7021). Cell lysis buffer was purchased from Cell Signalling Technology (#9803) and was supplemented with protease and phosphatase inhibitors (A32959; Thermo Fisher Scientific).

### 2.6 ELISAs, lactate dehydrogenase activity assay, and total protein content

Secreted interleukin‐6 (IL‐6), vascular cell adhesion molecule‐1 (VCAM‐1), endothelin (ET), and monocyte chemoattractant protein‐1 (MCP‐1) were measured in cell culture media by ELISA as per manufacturers' instructions (catalogue numbers DY206, DT809, DY1160, and DY279; R&D Systems). A lactate dehydrogenase (LDH) (Colorimetric) Assay Kit (category number ab102526; Abcam) was performed on cell media as per the manufacturer's instructions. A bicinchoninic acid protein assay was performed to quantify the total protein content in the cell lysates collected at the end of the experiments (P. K. Smith et al., 1985). A total osteocalcin ELISA which does not differentiate between uncarboxylated and carboxylated osteocalcin was performed to assess predicted and actual concentrations of ucOC added to experimental wells and to validate the purchased protein (DY1419; R&D Systems).

### 2.7 Statistical analysis

Statistical analyses were performed using GraphPad Prism (version 8.0 Graphpad Software Inc). Unpaired Student's *t* test was used to compare between the normal and atherogenic diet and between the NG and HG incubations in the ex vivo rabbit model. A one‐way analysis of variance (ANOVA) was used to analyse the effect of the ucOC treatment on blood vessel relaxation and immunohistochemistry staining. One‐way ANOVA was also used to detect differences between groups following the in vitro cell culture experiments. Post hoc analysis was completed using Fisher's least significance difference test. All data are reported as mean ± *SEM* and statistical analysis was conducted at the 95% level of significance (*p* < .05). Trends were reported if *p* was between 0.05 and 0.09. Effect sizes are commonly used to study the clinical relevance of intervention and show the magnitude of the effect that it is producing (Maylor, Zakrzewski‐Fruer, Stensel, Orton, & Bailey, 2019; Rodevand et al., 2019; Silva, Lacerda, & da Mota, 2019). The Cohen's *d* (*d*) equation was used to examine the magnitude of effect. A large effect is considered when *d* > 0.8, a medium effect between 0.5 and 0.79 and a small effect between 0.2 and 0.49 (Cohen, 2013).

## 3 RESULTS

### 3.1 Atherogenic diet versus normal diet in rabbits

**Figure 1:**
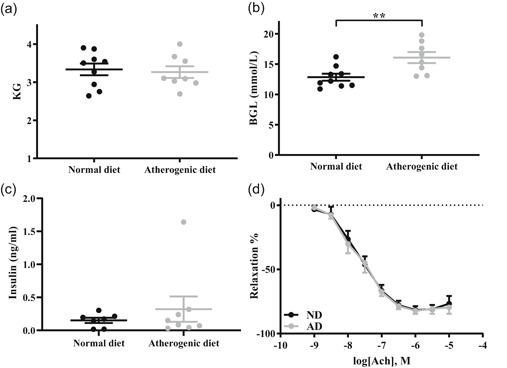
Comparison between the 4‐week normal and atherogenic diets for (a) Body mass, (b) random blood glucose concentration, (c) random insulin concentration, and (d) blood vessel relaxation. All data mean ± *SEM*. AD, atherogenic diet; BGL, blood glucose level; ND, normal diet. ** *p* < .01 between diets

No difference in body mass occurred between the rabbits fed a normal diet and those fed the atherogenic diet (*p* > .05, *d* = 0.16; Figure 1a). Circulating blood glucose levels was increased by 25% following the atherogenic diet compared to the normal diet (*p* < .01, *d* = 1.48; Figure 1b). Circulating insulin concentration did not change following both diets, suggesting the presence of insulin resistance in the animals fed with atherogenic diet (*p* > .05, *d* = 0.44; Figure 1c). Endothelial function was not altered by the atherogenic diet as shown by EC_50_, *E*_max_, and AUC (*p* > .05 for all, *d* = 0.03, 0.08 and 0.07, respectively; Figure 1d) compared to the normal diet.

### 3.2 Blood vessel reactivity

**Figure 2:**
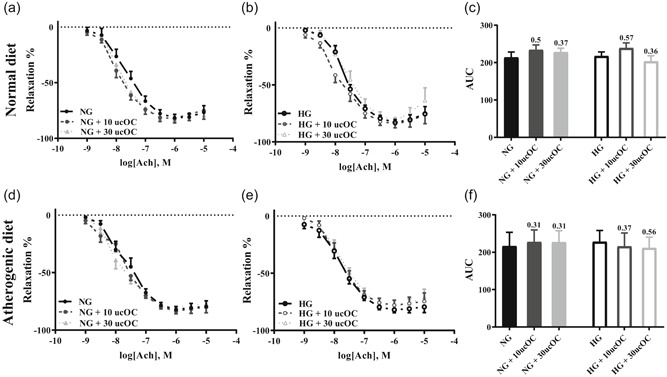
ACh‐induced endothelium‐dependent dose‐response curves in abdominal aorta following a 5‐min ucOC preincubation. ND (a–c) and AD (d–f) fed rabbits. All data mean ± *SEM*. Numbers above columns represent the effect size (Cohen's *d*) in comparison to the respective NG/HG control group. 10 ucOC, 10 ng/ml ucOC treatment; 30 ucOC, 30 ng/ml ucOC treatment; ACh, acetylcholine; AD, atherogenic diet; AUC, area under the curve; HG, high glucose media; ND, normal diet; NG, normal glucose media; ucOC, undercarboxylated osteocalcin

The 20 mM high‐glucose incubation did not alter endothelium‐dependent vasodilation following either the normal diet (*d* = EC_50_: 0.17; *E*_max_: 0.18) or atherogenic diet (*d* = EC_50_: 0.52; *E*_max_: 0.01; *p* > .05 for all; Figure S1). UcOC (10 and 30 ng/ml) did not alter ACh‐induced blood vessel relaxation in rabbits fed a normal or atherogenic diet or in aortic rings incubated in NG or HG solution (*p* > .05; Figure 2a-f; Table 1). The 10 ng/ml ucOC treatment produced a trend in Log EC_50_ (*p* = .05–.09; Table 1) and moderate improvements in AUC (∼10%; Figure 2c) as indicated by Cohen's *d* in NG and HG from normal diet‐fed rabbits. Administration of ucOC (10 and 30 ng/ml) before SNP induced endothelium‐independent relaxation did not significantly alter any measure of blood vessel relaxation (Figure S2 and Table S1).

**Table 1:** Log EC_50_ and *E*_max_ results from ACh‐induced endothelium‐dependent dose‐response curves in abdominal aorta

	*n*	Log EC_50_ ± SEM	*p* vs. NG/HG	*d* vs. NG/HG	E_max_ ± *SEM*	*p* vs. NG/HG	*d* vs. NG/HG
ND + NG	9	−7.67 ± 0.15			−81.54 ± 3.05		
ND + NG + 10 ng/ml ucOC	8	−7.96 ± 0.08	**.07^**	0.82	−82.46 ± 3.72	NS	0.09
ND + NG + 30 ng/ml ucOC	8	−7.88 ± 0.09	NS	0.58	−83.16 ± 2.4	NS	0.2
ND + HG	9	−7.73 ± 0.07			−83.1 ± 2.77		
ND + HG + 10 ng/ml ucOC	8	−7.96 ± 0.08	**.09^**	1.06	−84.21 ± 3.38	NS	0.12
ND + HG + 30 ng/ml ucOC	8	−7.59 ± 0.13	NS	0.46	−81.43 ± 2.61	NS	0.21
AD + NG	8	−7.68 ± 0.17			−82.18 ± 2.59		
AD + NG + 10 ng/ml ucOC	8	−7.76 ± 0.12	NS	0.18	−83.62 ± 2.15	NS	0.21
AD + NG + 30 ng/ml ucOC	8	−7.96 ± 0.12	NS	0.68	−80.3 ± 1.54	NS	0.31
AD + HG	7	−7.93 ± 0.18			−82.09 ± 2.56		
AD + HG + 10 ng/ml ucOC	7	−7.89 ± 0.12	NS	0.1	−78.08 ± 4.47	NS	0.42
AD + HG + 30 ng/ml ucOC	7	−7.74 ± 0.1	NS	0.49	−79.3 ± 1.94	NS	0.46

*Note*: 10 ucOC, 10 ng/ml ucOC treatment; 30 ucOC, 30 ng/ml ucOC treatment. ^*p* = .05–.099.

Abbreviations: ACh, acetylcholine; AD, atherogenic diet; *d*, Cohen's *d*; HG, high glucose media; *n*, total number of animals; ND, normal diet; NG, normal glucose media; NS, not significant; ucOC, undercarboxylated osteocalcin.

### 3.3 Immunohistochemistry

**Figure 3:**
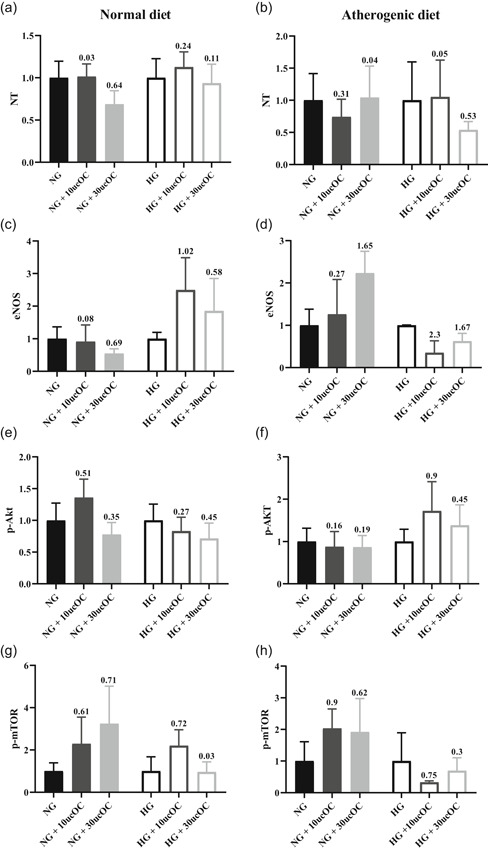
Immunoreactivity of (a and b) NT, (c and d) eNOS, (e and f) p‐AKT, and (g and h) p‐mTOR in aorta following normal diet (a,c,e,h) and atherogenic diet (b,d,g,i). Reactivity is calculated based on intensity of staining present on the endothelium which is an arbitrary unit and expressed as fold change from the respective NG/HG control group. Numbers above columns represent the effect size (Cohen's *d*) in comparison to the respective NG/HG control group. All data mean ± *SEM*. eNOS, endothelial nitric oxide synthase; HG, high glucose; NG, normal glucose; NT, nitrotyrosine; p‐Akt, phosphorylated protein kinase B; p‐mTOR, phosphorylated mammalian target of rapamycin; ucOC, undercarboxylated osteocalcin

The ucOC (10 and 30 ng/ml) treatment did not cause any significant changes in the immunoreactivity of NT, eNOS, p‐Akt, or p‐mTOR following either the normal or atherogenic diet (*p* > .05; Figure 3a–h). Analysis of Cohen's *d* revealed moderate to large increases in the reactivity of eNOS in HG incubated aorta following the normal diet (10 ng/ml ucOC = 2.5‐fold and 30 ng/ml ucOC 0.9‐fold) following ucOC administration. This was also found in the NG incubated aorta following the atherogenic diet (30 ng/ml ucOC = 1.2‐fold; Figure 3c,d). ucOC administration also increased the phosphorylation of mTOR at ser2448 in the NG condition following both normal (10 ng/ml ucOC = 1.3‐fold and 30 ng/ml ucOC = 2.2‐fold) and atherogenic diets (10 ng/ml ucOC = 1‐fold and 30 ng/ml = 0.9‐fold), while less effect occurred in the HG incubated vessels (Figure 3g,h).

### 3.4 Cell culture

**Figure 4:**
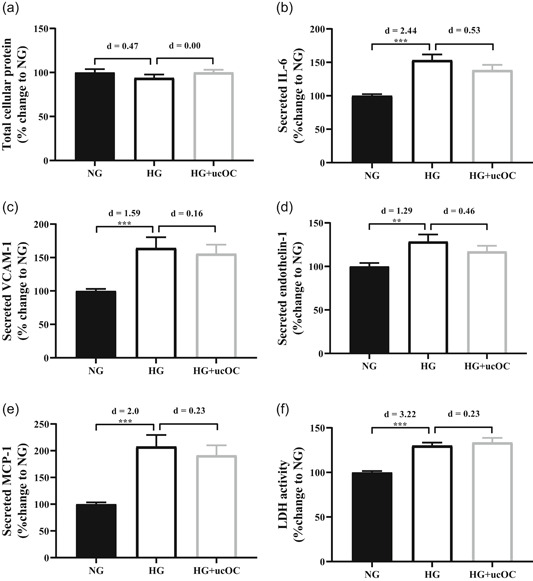
Human aortic endothelial cells (HAECs) cultured in NG media (NG; 5.5 mM) or HG media (HG; 16 mM) with or without ucOC (10 ng/ml). (a) Total protein content, (b) IL‐6, (c) VCAM‐1, (d) endothelin‐1, (e) MCP‐1 and (f) LDH activity were measured after 7 days. Numbers above columns represent the effect size (Cohen's *d*) in comparison to NG column. ** *p* < 0.01, *** *p* < .001 compared to NG. *n* = 12 for each condition from three experimental repeats. All data mean ± *SEM*. HG, high glucose; IL‐6, interleukin 6; LDH, lactate dehydrogenase; MCP‐1, monocyte chemoattractant protein‐1; NG, normal glucose; ucOC, undercarboxylated osteocalcin; VCAM‐1, vascular adhesion molecule 1

Total protein content was unaltered between the three experimental conditions (Figure 4a). After 7 days cultured in HG media (16 mM), the secretion of IL‐6, VCAM‐1, ET‐1, MCP‐1 and LDH were increased compared to NG controls (53%, 64%, 29%, 108% and 30%, respectively; *p* < .01 for all; Figure 4b–f). The addition of ucOC to HG media did not attenuate the increases in IL‐6, VCAM‐1, ET‐1, MCP‐1 or LDH activity (*p* > .05 for all). The HG + ucOC was also significantly increased compared to the NG controls for IL‐6, VCAM‐1, MCP‐1 and LDH (*p* < .01 for all). There was a trend for HG + ucOC to be elevated above the NG controls for ET‐1 (*p* = .07).

## 4 DISCUSSION

We report that acute ucOC treatment has no negative, or positive, effect on endothelial function or endothelial cell homeostasis in rabbit aorta ex vivo or human vascular cells in vitro in the presence or absence of high glucose.

Functionally, an impairment of endothelium‐dependent vasodilation is one of the first signs of endothelial dysfunction, and a marker of early atherosclerosis development (Bonetti et al., 2003; Esper et al., 2006). Previous research has examined endothelium‐dependent vasodilation in the thoracic aorta of apolipoprotein E (ApoE)−/− mice ex vivo following tOC administration. Following a 12‐week high‐fat diet with daily injections of vehicle or tOC (30 ng/g), the dilation of the thoracic aorta was improved by 20% in the tOC‐treated mice compared to the vehicle‐treated mice (Dou et al., 2014). However, the tOC‐treated mice also had improvements in body weight, blood glucose concentration, lipids and inflammatory markers (Dou et al., 2014). Therefore, whether improved blood vessel relaxation occurred as a direct response to OC or as a result of improved metabolic parameters cannot be determined from this study. Recently, ucOC was detectable within the endothelium of rabbit arteries, and administration of ucOC (10 ng/ml) improved endothelium‐dependent relaxation of the rabbit aorta (Qaradakhi et al., 2019). Suggesting that ucOC is present in the vasculature and can direct regulate blood vessel function.

As ucOC is known to exert a metabolic function (Ferron et al., 2008), we sought to determine if the effect of ucOC on blood vessel function remains under high glucose conditions. In the current study, the aortic sections from rabbits incubated for 2 h in 20 mM high glucose solution did not have altered endothelium‐dependent vasodilation, which is in contrast to previous studies (X. Guo et al., 2000; Taylor & Poston, 1994). Similarly, the atherogenic diet, which has previously been shown to cause endothelial dysfunction in rabbit aorta (Qaradakhi et al., 2019; Zulli & Hare, 2009), did not cause dysfunction in this study. It is unclear why in the endothelial dysfunction did not occur, but we were able to examine the effect of ucOC following acute high glucose incubations and in an insulin‐resistant state following the atherogenic diet. Whilst the administration of 10 ng/ml ucOC produced a trend towards an improvement in endothelium‐dependent relaxation, overall ucOC did not alter relaxation under any condition. This finding is in contrast to previous studies, but suggests that ucOC may not have a biological role in the regulation of endothelial function. Importantly, there was no indication of an adverse effect of ucOC on blood vessel function.

To examine the effect of ucOC on smooth muscle cells, independent of the endothelium, we administered ucOC to rabbit aorta before completing dose‐response curves with SNP, a nitric oxide donor. The acute ucOC treatment had no effect on SNP‐induced, endothelium‐independent relaxation of the rabbit aorta and suggests that any effect of ucOC in the vasculature is likely occurring via endothelium‐dependent mechanisms.

Previous in vivo experiments in mice demonstrated an improvement in endothelial relaxation following treatment with tOC (30 ng/ml) for 12 weeks. The authors suggested that the activation of the phosphatidylinositol 3‐kinase (PI3k)/Akt/eNOS signalling pathway was necessary to induce the enhancement in relaxation (Dou et al., 2014). In the current study, Akt and eNOS immunoreactivity was unaltered by treatment with ucOC. This is the same for the presence of NT and mTOR and suggests that the duration of ucOC treatment may not have been long enough to induce changes in the cellular signalling mechanisms necessary to alter endothelial function. Overall, in the current study, ucOC did not have a regulatory effect on the vasoactivity of rabbit aorta. Whilst the focus of this study was predominantly on vasodilation, the examination of the proposed signalling pathways that ucOC activates in endothelial cells did not reveal any changes following ucOC treatment. It is possible that a different dose of ucOC or a longer incubation is needed before ACh‐induced relaxation to cause a change in the signalling pathways or functional outcomes. This should be explored in future studies.

Endothelial cells have an important role in the maintenance of vascular homeostasis and the protection against the development of vascular disease (Brown, Shantsila, Varma, & Lip, 2017). Recent data suggest that ucOC may be involved in endothelial function. Administration of ucOC (25 and 100 ng/ml), but not the carboxylated form (cOC), increases eNOS phosphorylation in HAECs in a dose‐dependent manner (Kondo et al., 2016). Similarly, in HAEC incubation of ucOC for 1 h caused a dose‐dependent increase in eNOS phosphorylation at serine 1177. This was associated with dose‐dependent increases of Akt, an upstream activator of eNOS and of nitric oxide (Jung et al., 2013). Furthermore, increased eNOS, Akt and PI3k phosphorylation was reported in human umbilical cord vein endothelial cells following tOC (100 ng/ml) and ucOC (5 ng/ml) treatment (Dou et al., 2014; Q. Guo et al., 2017). Altogether, these studies support the hypothesis that OC, via ucOC, has an active role in endothelial cells, protecting against pathological processes and improving endothelial function via the PI3K/Akt signalling pathway (Tacey et al., 2018). However, we have recently reported some conflicting findings, ucOC treatment (10 ng/ml) did not alter the phosphorylation of Akt, mTOR, nuclear factor‐κB and several other markers of intracellular signalling in HAECs (Millar, Anderson, & O'Sullivan, 2019). In addition, ucOC did not alter markers of angiogenesis in HAEC, such as migration and matrix degradation and inflammatory markers that are commonly involved in endothelial dysfunction (Millar, Anderson et al., 2019). Furthermore, under acute and chronic inflammatory conditions that mimic an atherogenic environment, ucOC (10 ng/ml) had no anti‐inflammatory effect in human HAECs (Millar, Zala, et al., 2019). These results are supported by the findings of the current study. Here we show that ucOC administration did not attenuate inflammatory or dysfunction markers altered by high glucose treatment. One potential explanation for our finding is due to the dose of ucOC used. We used 10 ng/ml of ucOC, which is lower than what was used in some previous studies; however, it is in the physiological range (Hiam et al., 2019). Future research may complete a dose‐response curve to determine if there is an optimal dose of ucOC. Overall, our findings suggest that ucOC does not regulate endothelial cell signalling or function in physiological or pathophysiological conditions.

Although it was not examined in this study, there is evidence to suggest a link between OC and the advanced stages of atherosclerotic cardiovascular disease development, in particular the development of vascular calcification (Levinger et al., 2017). The mineralisation of plaque during atherosclerosis development is similar to the formation of bone within the skeleton (Zhu, Mackenzie, Farquharson, & Macrae, 2012). The form of OC present within calcified plaques is yet to be identified, given the role of cOC in the mineralisation of bone it is possible that cOC is responsible for the association with vascular calcification. The exact mechanisms by which OC mediates the interaction with vascular calcification is still to be fully identified, but should be investigated in future studies.

A limitation of this study is that the atherogenic diet and the 20 mM glucose incubation which have previously been shown to cause endothelial dysfunction, did not alter endothelial function, indicating heterogeneity of blood vessel dysfunction. However, we were still able to assess the effect of ucOC on endothelial function in normal and high glucose environments.

In conclusion, acute ucOC treatment does not have a negative, or positive, effect on endothelial function or endothelial cell homeostasis in rabbit aorta or human vascular cells in the presence or absence of a high glucose environment. As no adverse effects occurred, it is proposed that ucOC may be considered as a therapeutic option for metabolic disease.

## CONFLICT OF INTERESTS

The authors declare that there are no conflict of interests.

## AUTHOR CONTRIBUTIONS

The research was designed by Alexander Tacey, Sophie Millar, Susan Anderson, Anthony Zulli, Saoirse O'Sullivan and Itamar Levinger. The experiments were completed by Alexander Tacey, Sophie Millar and Tawar Qaradakhi. The original draft was written by Alexander Tacey. The manuscript was reviewed and edited by Sophie Millar, Tawar Qaradakhi, Cassandra Smith, Alan Hayes, Susan Anderson, Anthony Zulli, Saoirse O'Sullivan and Itamar Levinger. All authors approved the final version of the manuscript.

## Supporting information

Supporting information.

Supporting information.

Supporting information.

## Data Availability

The data from this study are available upon reasonable request.

## References

[jcp30048-bib-0001] Arora, R. , Hare, D. L. , & Zulli, A. (2012). Simvastatin reduces endothelial NOS: caveolin‐1 ratio but not the phosphorylation status of eNOS in vivo. Journal of Atherosclerosis and Thrombosis, 19(8), 705–711. 22850448

[jcp30048-bib-0002] Bakker, W. , Eringa, E. C. , Sipkema, P. , & van Hinsbergh, V. W. (2009). Endothelial dysfunction and diabetes: Roles of hyperglycemia, impaired insulin signaling and obesity. Cell and Tissue Research, 335(1), 165–189. 10.1007/s00441-008-0685-6 18941783

[jcp30048-bib-0003] Bonetti, P. O. , Lerman, L. O. , & Lerman, A. (2003). Endothelial dysfunction: A marker of atherosclerotic risk. Arteriosclerosis, Thrombosis, and Vascular Biology, 23(2), 168–175. 10.1161/01.atv.0000051384.43104.fc 12588755

[jcp30048-bib-0004] Bornfeldt, K. E. , & Tabas, I. (2011). Insulin resistance, hyperglycemia, and atherosclerosis. Cell Metabolism, 14(5), 575–585. 10.1016/j.cmet.2011.07.015 22055501 PMC3217209

[jcp30048-bib-0005] Brown, R. A. , Shantsila, E. , Varma, C. , & Lip, G. Y. (2017). Current understanding of atherogenesis. American Journal of Medicine, 130(3), 268–282. 10.1016/j.amjmed.2016.10.022 27888053

[jcp30048-bib-0006] Cahill, P. A. , & Redmond, E. M. (2016). Vascular endothelium—Gatekeeper of vessel health. Atherosclerosis, 248, 97–109. 10.1016/j.atherosclerosis.2016.03.007 26994427 PMC6478391

[jcp30048-bib-0007] Cohen, J. (2013). Statistical power analysis for the behavioral sciences. Abingdon, UK: Routledge.

[jcp30048-bib-0008] Dou, J. , Li, H. , Ma, X. , Zhang, M. , Fang, Q. , Nie, M. , … Jia, W. (2014). Osteocalcin attenuates high fat diet‐induced impairment of endothelium‐dependent relaxation through Akt/eNOS‐dependent pathway. Cardiovascular Diabetology, 13, 74 10.1186/1475-2840-13-74 24708830 PMC4233640

[jcp30048-bib-0009] Ebong, I. A. , Goff, D. C., Jr. , Rodriguez, C. J. , Chen, H. , Sibley, C. T. , & Bertoni, A. G. (2013). Association of lipids with incident heart failure among adults with and without diabetes mellitus: Multiethnic study of atherosclerosis. Circulation: Heart Failure, 6(3), 371–378. 10.1161/circheartfailure.112.000093 23529112 PMC3991930

[jcp30048-bib-0010] El‐Hawli, A. , Qaradakhi, T. , Hayes, A. , Rybalka, E. , Smith, R. , Caprnda, M. , … Zulli, A. (2017). IRAP inhibition using HFI419 prevents moderate to severe acetylcholine mediated vasoconstriction in a rabbit model. Biomedicine & Pharmacotherapy, 86, 23–26. 10.1016/j.biopha.2016.11.142 27936390

[jcp30048-bib-0011] Esper, R. J. , Nordaby, R. A. , Vilarino, J. O. , Paragano, A. , Cacharron, J. L. , & Machado, R. A. (2006). Endothelial dysfunction: A comprehensive appraisal. Cardiovascular Diabetology, 5, 4 10.1186/1475-2840-5-4 16504104 PMC1434727

[jcp30048-bib-0012] Ferron, M. , Hinoi, E. , Karsenty, G. , & Ducy, P. (2008). Osteocalcin differentially regulates beta cell and adipocyte gene expression and affects the development of metabolic diseases in wild‐type mice. Proceedings of the National Academy of Sciences of the United States of America, 105(13), 5266–5270. 10.1073/pnas.0711119105 18362359 PMC2278202

[jcp30048-bib-0013] Guo, Q. , Li, H. , Xu, L. , Wu, S. , Sun, H. , & Zhou, B. (2017). Undercarboxylated osteocalcin reverts insulin resistance induced by endoplasmic reticulum stress in human umbilical vein endothelial cells. Scientific Reports, 7(1):46 10.1038/s41598-017-00163-2 28246389 PMC5427815

[jcp30048-bib-0014] Guo, X. , Liu, W. L. , Chen, L. W. , & Guo, Z. G. (2000). High glucose impairs endothelium‐dependent relaxation in rabbit aorta. Acta Pharmacologica Sinica, 21(2), 169–173. 11263266

[jcp30048-bib-0015] Hiam, D. , Voisin, S. , Yan, X. , Landen, S. , Jacques, M. , Papadimitriou, I. D. , … Eynon, N. (2019). The association between bone mineral density gene variants and osteocalcin at baseline, and in response to exercise: The gene SMART study. Bone, 123, 23–27. 10.1016/j.bone.2019.03.015 30878522

[jcp30048-bib-0016] Jung, C. H. , Lee, W. J. , Hwang, J. Y. , Lee, M. J. , Seol, S. M. , Kim, Y. M. , … Park, J. Y. (2013). The preventive effect of uncarboxylated osteocalcin against free fatty acid‐induced endothelial apoptosis through the activation of phosphatidylinositol 3‐kinase/Akt signaling pathway. Metabolism: Clinical and Experimental, 62(9), 1250–1257. 10.1016/j.metabol.2013.03.005 23639572

[jcp30048-bib-0017] Kanazawa, I. , Yamaguchi, T. , Yamamoto, M. , Yamauchi, M. , Kurioka, S. , Yano, S. , & Sugimoto, T. (2009). Serum osteocalcin level is associated with glucose metabolism and atherosclerosis parameters in type 2 diabetes mellitus. Journal of Clinical Endocrinology and Metabolism, 94(1), 45–49. 10.1210/jc.2008-1455 18984661

[jcp30048-bib-0018] Kanazawa, I. , Yamaguchi, T. , Yamauchi, M. , Yamamoto, M. , Kurioka, S. , Yano, S. , & Sugimoto, T. (2011). Serum undercarboxylated osteocalcin was inversely associated with plasma glucose level and fat mass in type 2 diabetes mellitus. Osteoporosis International, 22(1), 187–194. 10.1007/s00198-010-1184-7 20165834

[jcp30048-bib-0019] Kondo, A. , Kawakubo‐Yasukochi, T. , Mizokami, A. , Chishaki, S. , Takeuchi, H. , & Hirata, M. (2016). Uncarboxylated osteocalcin increases serum nitric oxide levels and ameliorates hypercholesterolemia in mice fed an atherogenic diet. Electronic Journal of Biology, 13, 1.

[jcp30048-bib-0020] Lerman, A. , & Zeiher, A. M. (2005). Endothelial function: Cardiac events. Circulation, 111(3), 363–368. 10.1161/01.CIR.0000153339.27064.14 15668353

[jcp30048-bib-0021] Levinger, I. , Brennan‐Speranza, T. C. , Zulli, A. , Parker, L. , Lin, X. , Lewis, J. R. , & Yeap, B. B. (2017). Multifaceted interaction of bone, muscle, lifestyle interventions and metabolic and cardiovascular disease: Role of osteocalcin. Osteoporosis International, 28(8), 2265–2273. 10.1007/s00198-017-3994-3 28289780

[jcp30048-bib-0022] Li, J. , Zhang, H. , Yang, C. , Li, Y. , & Dai, Z. (2016). An overview of osteocalcin progress. Journal of Bone and Mineral Metabolism, 34(4), 367–379. 10.1007/s00774-015-0734-7 26747614

[jcp30048-bib-0023] Lin, X. , Parker, L. , McLennan, E. , Zhang, X. , Hayes, A. , McConell, G. , … Levinger, I. (2017). Recombinant uncarboxylated osteocalcin per se enhances mouse skeletal muscle glucose uptake in both extensor digitorum longus and soleus muscles. Frontiers in Endocrinology, 8, 330 10.3389/fendo.2017.00330 29204135 PMC5698688

[jcp30048-bib-0024] Maylor, B. D. , Zakrzewski‐Fruer, J. K. , Stensel, D. J. , Orton, C. J. , & Bailey, D. P. (2019). Effects of Frequency and duration of interrupting sitting on cardiometabolic risk markers. International Journal of Sports Medicine, 40, 818–824. 10.1055/a-0997-6650 31499563

[jcp30048-bib-0025] Millar, S. A. , Anderson, S. I. , & O'Sullivan, S. E. (2019). Human vascular cell responses to the circulating bone hormone osteocalcin. Journal of Cellular Physiology, 234(11), 21039–21048. 10.1002/jcp.28707 31026070 PMC6767466

[jcp30048-bib-0026] Millar, S. A. , Patel, H. , Anderson, S. I. , England, T. J. , & O'Sullivan, S. E. (2017). Osteocalcin, vascular calcification, and atherosclerosis: A systematic review and meta‐analysis. Front Endocrinol (Lausanne), 8, 183 10.3389/fendo.2017.00183 28824544 PMC5534451

[jcp30048-bib-0027] Millar, S. A. , Zala, I. , Anderson, S. I. , & O'Sullivan, S. E. (2019). Osteocalcin does not influence acute or chronic inflammation in human vascular cells. Journal of Cellular Physiology, 235, 3414–3424. 10.1002/jcp.29231 31549398 PMC6972510

[jcp30048-bib-0028] Oury, F. , Sumara, G. , Sumara, O. , Ferron, M. , Chang, H. , Smith, C. E. , … Karsenty, G. (2011). Endocrine regulation of male fertility by the skeleton. Cell, 144(5), 796–809. 10.1016/j.cell.2011.02.004 21333348 PMC3052787

[jcp30048-bib-0029] Qaradakhi, T. , Gadanec, L. K. , Tacey, A. B. , Hare, D. L. , Buxton, B. F. , Apostolopoulos, V. , … Zulli, A. (2019). The effect of recombinant undercarboxylated osteocalcin on endothelial dysfunction. Calcified Tissue International, 105(5), 546–556. 10.1007/s00223-019-00600-6 31485687

[jcp30048-bib-0030] Qaradakhi, T. , Matsoukas, M. T. , Hayes, A. , Rybalka, E. , Caprnda, M. , Rimarova, K. , … Zulli, A. (2017). Alamandine reverses hyperhomocysteinemia‐induced vascular dysfunction via PKA‐dependent mechanisms. Cardiovascular Therapeutics, 35(6), e12306 10.1111/1755-5922.12306 28901725

[jcp30048-bib-0031] Rask‐Madsen, C. , & King, G. L. (2013). Vascular complications of diabetes: Mechanisms of injury and protective factors. Cell Metabolism, 17(1), 20–33. 10.1016/j.cmet.2012.11.012 23312281 PMC3546345

[jcp30048-bib-0032] Reyes‐Garcia, R. , Rozas‐Moreno, P. , Jimenez‐Moleon, J. J. , Villoslada, M. J. , Garcia‐Salcedo, J. A. , Santana‐Morales, S. , & Munoz‐Torres, M. (2012). Relationship between serum levels of osteocalcin and atherosclerotic disease in type 2 diabetes. Diabetes & Metabolism, 38(1), 76–81. 10.1016/j.diabet.2011.07.008 21996253

[jcp30048-bib-0033] Rodevand, L. , Steen, N. E. , Elvsashagen, T. , Quintana, D. S. , Reponen, E. J. , Morch, R. H. , … Andreassen, O. A. (2019). Cardiovascular risk remains high in schizophrenia with modest improvements in bipolar disorder during past decade. Acta Psychiatrica Scandinavica, 139(4), 348–360. 10.1111/acps.13008 30697685

[jcp30048-bib-0034] Silva, A. S. E. , Lacerda, F. V. , & da Mota, M. P. G. (2019). Effect of strength training on plasma levels of homocysteine in patients with type 2 diabetes. International Journal of Preventive Medicine, 10, 80 10.4103/ijpvm.IJPVM_313_17 31198515 PMC6547780

[jcp30048-bib-0035] Smith, P. K. , Krohn, R. I. , Hermanson, G. T. , Mallia, A. K. , Gartner, F. H. , Provenzano, M. D. , … Klenk, D. C. (1985). Measurement of protein using bicinchoninic acid. Analytical Biochemistry, 150(1), 76–85. 10.1016/0003-2697(85)90442-7 3843705

[jcp30048-bib-0036] Smith, R. M. , Rai, S. , Kruzliak, P. , Hayes, A. , & Zulli, A. (2019). Putative Nox2 inhibitors worsen homocysteine‐induced impaired acetylcholine‐mediated relaxation. Nutrition, Metabolism, and Cardiovascular Diseases, 29(8), 856–864. 10.1016/j.numecd.2019.05.051 31272869

[jcp30048-bib-0037] Tacey, A. , Qaradakhi, T. , Brennan‐Speranza, T. , Hayes, A. , Zulli, A. , & Levinger, I. (2018). Potential role for osteocalcin in the development of atherosclerosis and blood vessel disease. Nutrients, 10(10):1426 10.3390/nu10101426 PMC6213520 30287742

[jcp30048-bib-0038] Taylor, P. D. , & Poston, L. (1994). The effect of hyperglycaemia on function of rat isolated mesenteric resistance artery. British Journal of Pharmacology, 113(3), 801–808. 7858870 10.1111/j.1476-5381.1994.tb17064.x PMC1510412

[jcp30048-bib-0039] Villafan‐Bernal, J. R. , Sanchez‐Enriquez, S. , & Munoz‐Valle, J. F. (2011). Molecular modulation of osteocalcin and its relevance in diabetes (Review). International Journal of Molecular Medicine, 28(3), 283–293. 10.3892/ijmm.2011.706 21617842

[jcp30048-bib-0040] Zhu, D. , Mackenzie, N. C. , Farquharson, C. , & Macrae, V. E. (2012). Mechanisms and clinical consequences of vascular calcification. Front Endocrinol, 3, 95 10.3389/fendo.2012.00095 PMC3412412 22888324

[jcp30048-bib-0041] Zulli, A. , Buxton, B. F. , Black, M. J. , Ming, Z. , Cameron, A. , & Hare, D. L. (2006). The immunoquantification of caveolin‐1 and eNOS in human and rabbit diseased blood vessels. Journal of Histochemistry and Cytochemistry, 54(2), 151–159. 10.1369/jhc.5A6677.2005 16009963

[jcp30048-bib-0042] Zulli, A. , & Hare, D. L. (2009). High dietary methionine plus cholesterol stimulates early atherosclerosis and late fibrous cap development which is associated with a decrease in GRP78 positive plaque cells. International Journal of Experimental Pathology, 90(3), 311–320. 10.1111/j.1365-2613.2009.00649.x 19563613 PMC2697553

