# Supplementary material for: Undercarboxylated osteocalcin has no adverse effect on endothelial function in rabbit aorta or human vascular cells
Source: J Cell Physiol. 2020 Sep 16;236(4):2840–9. doi: 10.1002/jcp.30048 (PMC7891339; doi:10.1002/jcp.30048)
Supplement: Supplementary file 3 — Supporting information. [file JCP-236-2840-s003.docx]

**Supplementary Table 1** Log EC_50_ and E_max_ results from SNP-induced endothelium-independent dose response curves in abdominal aorta.

|  | n | Log EC_50_ ± SEM | p vs NG/HG | d vs NG/HG | E_max_ ± SEM | p vs NG/HG | d vs NG/HG |
| --- | --- | --- | --- | --- | --- | --- | --- |
| ND + NG | 9 | -7.65 ± 0.11 |  |  | -93.2 ± 1.12 |  |  |
| ND + NG + 10ng/ml ucOC | 8 | -7.66 ± 0.14 | n/s | 0.02 | -94.68 ± 0.88 | n/s | 0.5 |
| ND + NG + 30ng/ml ucOC | 8 | -7.67 ± 0.15 | n/s | 0.06 | -94.6 ± 1.47 | n/s | 0.37 |
| ND + HG | 8 | -7.69 ± 0.1 |  |  | -92.13 ± 1.33 |  |  |
| ND + HG + 10ng/ml ucOC | 8 | -7.88 ± 0.17 | n/s | 0.48 | -94.05 ± 1.57 | n/s | 0.47 |
| ND + HG + 30ng/ml ucOC | 8 | -7.63 ± 0.15 | n/s | 0.17 | -94.98 ± 1.57 | n/s | 0.69 |
| AD + NG | 7 | -7.6 ± 0.16 |  |  | -92.98 ± 0.66 |  |  |
| AD + NG + 10ng/ml ucOC | 7 | -7.62 ± 0.13 | n/s | 0.06 | -91.47 ± 1.08 | n/s | 0.64 |
| AD + NG + 30ng/ml ucOC | 7 | -7.58 ± 0.15 | n/s | 0.06 | -94.24 ± 1.4 | n/s | 0.44 |
| AD + HG | 6 | -7.69 ± 0.16 |  |  | -93.36 ± 1.07 |  |  |
| AD + HG + 10ng/ml ucOC | 6 | -7.78 ± 0.24 | n/s | 0.18 | -92.53 ± 2.02 | n/s | 0.21 |
| AD + HG + 30ng/ml ucOC | 6 | -7.74 ± 0.19 | n/s | 0.11 | -89.34 ± 0.72 | **0.06^** | 1.8 |

*Abbreviations: 10ucOC; 10ng/ml ucOC treatment, 30ucOC; 30ng/ml ucOC treatment, AD; atherogenic diet, d; Cohen’s d, HG; high glucose media, ND; normal diet, NG; normal glucose media, n; total number of animals.*

**Supplementary Figure 1** ACh-induced endothelium-dependent dose response curves in abdominal aorta. ND (A + C) and AD (B + C) fed rabbits. All data mean ± SEM. Numbers above columns represent the effect size (Cohen’s d) in comparison to the respective NG control group.
Abbreviations: *Ach; acetylcholine, AD; atherogenic diet, HG; high glucose media, ND; normal diet, NG; normal glucose media.*

**Supplementary Figure 2** SNP-induced endothelium-independent dose response curves in abdominal aorta following a 5-minute ucOC pre-incubation. ND (A-C) and AD (D-F) fed rabbits. All data mean ± SEM. Numbers above columns represent the effect size (Cohen’s d) in comparison to the respective NG/HG control group.
Abbreviations: *10ucOC; 10ng/ml ucOC treatment, 30ucOC; 30ng/ml ucOC treatment, AD; atherogenic diet, AUC; area under the curve; HG; high glucose media, ND; normal diet, NG; normal glucose media, SNP; sodium nitroprusside.*
